# Long term survival in a case of concurrent retroperitoneal liposarcoma and renal cell carcinoma: a case report

**DOI:** 10.1186/1756-0500-7-538

**Published:** 2014-08-16

**Authors:** Senji Hoshi, Natuho Hayashi, Mayu Yagi, Teppei Ookubo, Akinori Muto, Osamu Sugano, Kenji Numahata, Vladimir Bilim, Kiyotugu Hoshi, Isoji Sasagawa, Syun-ichi Saso

**Affiliations:** Department of Urology, Yamagata Prefectural Central Hospital, Yamagata, Japan; Department of Urology, Niigata Cancer Center Hospital, Kawagishi-cho 2-15-3, Chuo-ku, Niigata-shi, 951-8566 Japan; Department of Urology, Yamagata Tokushukai Hospital, Yamagata, Japan; Department of Pathology, Hachinohe Japan Red Cross Hospital, Hachinohe, Japan

**Keywords:** Retroperitoneal liposarcoma, Renal cell carcinoma

## Abstract

**Background:**

Liposarcoma is one of the most common soft tissue sarcomas found in adults. It has a predilection for retroperitoneal space. Renal cell carcinoma is the most common tumor of the kidney.

**Case presentation:**

Concurrent retroperitoneal liposarcoma and renal cell carcinoma were found in a 34-year-old Japanese man. The renal tumor was first detected by ultrasonography, it was confirmed by computed tomography, which also identified a presumptive retroperitoneal liposarcoma, and the tumors were further assessed with magnetic resonance imaging. The patient was treated by surgical resection of retroperitoneal liposarcoma and left nephrectomy and has been disease-free for 10 years.

**Conclusions:**

The concomitant occurrence of a renal tumor and a primary primary liposarcoma is rare. The major factors promoting a good prognosis in this case were the favorable histology and the small size of the tumors.

## Background

Liposarcoma is one of the most common soft tissue sarcomas found in adults, and it usually occurs in the retroperitoneum and the extremities [1]. Its incidence rate increases in middle-aged and older adults with a peak in 70–80 year olds. Renal cell carcinoma (RCC) is the most common tumor of the kidney. Its incidence is highest in those aged 50–70 years. The co-occurence of RCC and hematological malignancies as well as solid tumors has been reported.

## Case presentation

**Figure 1:**
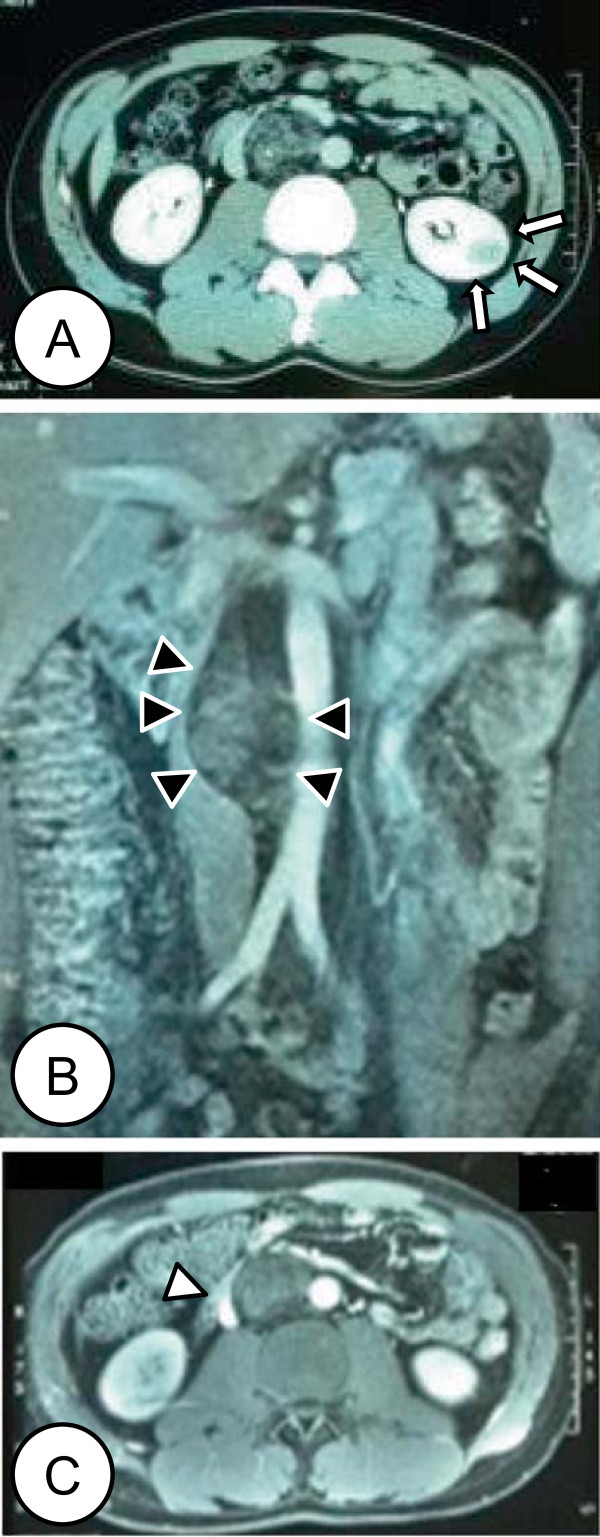
**Comuted tomography (CT) and magnetic resonance imaging (MRI) of the tumors.** Left lower pole renal tumor (indicated by arrows) and 5x4x7.5 cm interaortocaval tumor containing fatty tissue, calcification and soft tissue components (indicated by filled arrowheads). The inferior vena cava is compressed by the ineraortocaval tumor (indicated by open arrowhead). **(A)** Contrast-enhanced CT, parenchymal phase. Wash-out of the contrast is typical for clear cell RCC. Coronal **(B)** and axial **(C)** contrast-enhanced MRI.

**Figure 2:**
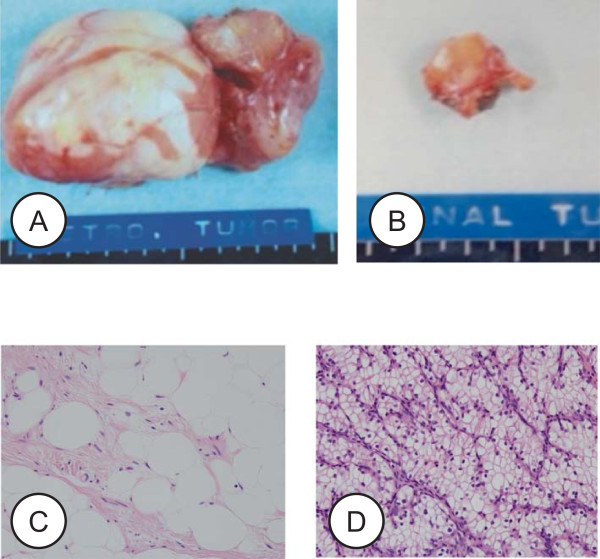
**Macro- and microphotograph of the resected tumors.** Resected retroperitoneal tumor **(A)** and kidney tumor **(B)**. Microphotograph of hematoxylin & eosin stained tissue section of the retroperitoneal tumor **(C)** represents the picture of a typical well-differentiated liposarcoma (X40). Microphotograph of hematoxylin & eosin stained tissue section of kidney tumor **(D)** represents clear cell RCC (X40).

A 34-year-old Japanese man, with no significant previous medical history, presented to our hospital because of an incidentally detected renal tumor. The patient did not have a family history of RCC or any signs of hereditary RCC syndrome on examination. He did not have any physical and laboratory findings indicating RCC including microscopic hematuria. Contrast enhanced computed tomography (CT) (Figure 1A) confirmed a 2.5 cm left kidney tumor; it showed the early enhancement and washout typical of a clear cell RCC. Additionally, a retroperitoneal tumor with calcification was identified, the presence of lipid and soft tissue components was confirmed, and a presumptive diagnosis of retroperitoneal liposarcoma was made (Figure 1A). Abdominal magnetic resonance imaging (MRI) (Figure 1B, C) showed a tumor located in the interaortocaval space with high signal intensity on the T2-weighted images. No apparent metastases were identified.Left partial nephrectomy and resection of the retroperitoneal tumor were performed in September 2003. The pathological diagnoses were clear cell renal carcinoma and retroperitoneal liposarcoma (Figure 2). The excised retroperitoneal tumor was a 6.8 × 4.8 cm well-circumscribed mass. Cut sections of the tumor had a lobulated yellowish appearance. Histological examination revealed the tumor to be composed of a mixture of fibrous tissue and mature-appearing adipose tissues (Figure 2C), with the fibrous tissue separation the adipose tissue into regions of varied size. The adipose cells appeared almost mature, but the tumor contained atypical cells with varied size and shape, including a few mono- or mutli-vacuolated lipoblastic cells. The fibrous tissues contained muscle fibers. Few cells were positive for MIB-1 antibody (proliferating cells). The resected renal tumor was 1.6 × 1.6 cm in size, histopathology showed a clear cell RCC (Figure 2D) circumscribed by a fibrous capsule, classified as G1 pT1a (i.e. less than 4 cm in size and confined to the kidney). Surgical margins of both tumors were negative and no adjuvant treatment was performed. Ten years after the operation, the patient is doing well and has not experienced a recurrence.

Young patient age can indicate a hereditary RCC syndrome. However, our patient had no family history and clinical signs of tuberous sclerosis, von Hippel-Lindau disease, and succinate dehydrogenase-associated familial cancer.

We identified five reported cases of concurrent liposarcoma and RCC [2–6] (Table 1). Patients age ranged from 58 to 79 years (median 71 years), slightly older than the average age for all RCC patients. The RCC histological subtype was clear cell in 1 case [4], papillary cell in 3 cases [2, 3, 6] and granular cell in 1 case [5] (note: it has recently been shown that “granular cell” type RCC is not an independent histological type). The location of liposarcoma was perirenal [4, 5] in 2 cases, retroperitoneal [2, 6] in 2 cases, and cardiac [3] in 1 case (found by autopsy). Although the number of reported cases is small, it is interesting to note that only one case represented the clear cell subtype whereas three cases were diagnosed as papillary cell RCC. In the present case retroperitoneal liposarcoma was located in interaortocaval space and RCC histology was clear cell. Our case and all of the previously reported cases were males, which fits with the male predisposition for RCC.

**Table 1:** **Summary of the five reported cases of concurrent liposarcoma associated with renal cell carcinoma (RCC)**

Reference	Year reported	Age (years)	RCC histological subtype	RCC size	RCC laterality	Liposarcoma location	Liposarcoma size	Recurrence
[2]	2013	74	Papillary	1.1 × 1.1 × 0.4 cm	Left	Retroperitoneum	13.8 × 15.2 cm	No data
[3]	2003	58	Papillary	1.0 cm	Left	Cardiac (found by autopsy)	8.0 cm	NA
[4]	2009	60	Clear cell	5.4 × 5.2 cm	Right	Perirenal	7.6 × 5.0 cm	NED 24 months
[5]	1994	71	Granular cell	4 cm	Right	Perirenal	No data	17 months no recurrence
[6]	2008	79	Papillary	0.55 cm (found incidentally)	Left	Retroperitoneum (invasion of the proximal ureter)	40 cm, 20 cm and 14 cm	Recurrent liposarcoma, no data on follow up

Abbreviations: *NA* not applicable, *NED* no evidence of disease, *RCC* renal cell carcinoma.

In addition to the previously described cases, we found two published case reports regarding liposarcomatous differentiation in chromophobe RCCs [7, 8]. In contrast, the renal tumor in our case was a typical clear cell RCC, and the two tumors represented distinct entities.

Over the last 15 years, 1123 patients with retroperitoneal soft tissue sarcoma have been reported in 25 series; these tumors had a mean diameter of 15.7 cm [9]. Retroperitoneal soft tissue sarcomas represent 0.10 to 0.15% of all malignancies and 45% of all retroperitoneal tumors. Because of the localization, symptoms are nonspecific (e.g., abdominal discomfort and palpable mass) and caused by tumor growth, which is typically very large when detected. The only curative treatment modality is complete surgical resection; chemotherapy and radiation therapy show no survival benefit. It has been reported that 51.4% of these tumors can be completely excised and that 50.2% of these excisions include adjacent organs [9]. The prognosis without complete excision is poor with reported 5- and 10-year survival rates of 16.7% and 8.0% respectively [9].

Local recurrence represents the major type of progression for retroperitoneal liposarcomas. Yamamoto *et al.* [10] described 45 patients with well-differentiated liposarcoma who underwent surgical treatment. Among 41 patients who underwent initial surgery, only one recurrence occurred, which was localized in the retroperitoneal space. For 4 patients who underwent a reoperation, the mean time between the initial surgery and the recurrence was 16.5 years. None of the 45 patients developed distant metastasis. In our case, during the 10 years of follow-up to date, no recurrence or metastasis has been detected. However, continued follow-up is necessary because late recurrences are common with liposarcoma.

Previously reported liposarcomas have demonstrated heterogeneous signal intensity on MRI with great variation depending on the components and histological patterns of a particular tumor. Retroperitoneal liposarcomas have been classified into several clinico-pathological subtypes [11]. Myxoid liposarcoma, consisting of a myxoid matrix and a small amount of mature fat, shows low signal intensity on T1 weighted image and high signal intensity on T2 weighted image [11]. Well-differentiated liposarcoma presented high signal intensity on T1 weighted images, intermediate signal intensity on T2 weighted images, drop-out signal intensity on fat-suppressed MR images [11]. Round-cell liposarcoma and pleomorphic liposarcoma exhibit the signal intensity of a soft-tissue tumor without a characteristic fat signal [11]. Liposarcomas can present with intratumoral hemorrhage and may invade adjacent organs. In the present case, the tumor showed high signal intensity on the T2-weighted images, which is typical for myxoid liposarcoma and is inconsistent with well-differentiated liposarcoma which was diagnosed pathologically. Five-year and ten-year disease specific survival is the highest for well-differentiated liposarcoma (100% and 87%) followed by myxoid liposarcoma (88% and 76%), and is the lowest for pleomorphic liposarcoma (56% and 39%) (http://sarcomahelp.org/liposarcoma.html).

## Conclusion

We have presented a case of concomitant RCC and retroperitoneal liposarcoma in a young male. Although no data exists regarding an association between these two malignant tumors, a genetic predisposition for cancer is likely present given patient’s young age. Both tumors were completely surgically excised and no relapse has been seen during ten-year follow-up to date. The major factors promoting a good prognosis in this case were favorable histology and small size of the tumors at initial diagnosis.

## Consent

Written informed consent was obtained from the patient for publication of this Case Report and any accompanying images. A copy of the written consent is available for review by the Editor-in-Chief of this journal.

## References

[1] Tsuruta A, Notohara K, Park T, Itoh T (2012). Dedifferentiated liposarcoma of the rectum: a case report. World J Gastroenterol.

[2] Frank RM, Velasco JM (2013). Surgical management of incidental renal tumor during excision of retroperitoneal liposarcoma and osteogenic sarcoma. Am Surg.

[3] Galazka K, Ciezarek M, Soja J, Krzanowski M, Szlubowski A, Sydor K, Adamczyk W, Grodecki J, Sladek K (2003). Synchronous primary heart liposarcoma and papillary renal carcinoma–a case report. Pol J Pathol.

[4] Kinebuchi Y, Ishizuka O, Minagawa T, Nisizawa O, Shimojo H (2009). Concurrent perirenal liposarcoma associated with renal cell carcinoma. Hinyokika Kiyo.

[5] Lewis DJ, Moul JW, Williams SC, Sesterhenn IA, Colon E (1994). Perirenal liposarcoma containing extramedullary hematopoiesis associated with renal cell carcinoma. Urology.

[6] Williamson JM, Konig TC, Canelo R (2008). Incidental finding of renal cell carcinoma in recurrent retroperitoneal liposarcoma. Ann R Coll Surg Engl.

[7] Anila KR, Mathew AP, Somanathan T, Mathews A, Jayasree K (2011). Chromophobe renal cell carcinoma with heterologous (Liposarcomatous) differentiation: a case report. Int J Surg Pathol.

[8] Petersson F, Michal M, Franco M, Hes O (2010). Chromophobe renal cell carcinoma with liposarcomatous dedifferentiation - report of a unique case. Int J Clin Exp Pathol.

[9] Bradley JC, Caplan R (2002). Giant retroperitoneal sarcoma: a case report and review of the management of retroperitoneal sarcomas. Am Surg.

[10] Yamamoto N, Hayashi K, Tanzawa Y, Kimura H, Takeuchi A, Igarashi K, Inatani H, Shimozaki S, Kitamura S, Tsuchiya H (2012). Treatment strategies for well-differentiated liposarcomas and therapeutic outcomes. Anticancer Res.

[11] Song T, Shen J, Liang BL, Mai WW, Li Y, Guo HC (2007). Retroperitoneal liposarcoma: MR characteristics and pathological correlative analysis. Abdom Imaging.

